# Lectins from the Edible Mushroom *Agaricus bisporus* and Their Therapeutic Potentials

**DOI:** 10.3390/molecules25102368

**Published:** 2020-05-20

**Authors:** Wangsa Tirta Ismaya, Raymond Rubianto Tjandrawinata, Heni Rachmawati

**Affiliations:** 1 Dexa Laboratories of Biomolecular Sciences, Dexa Medica, Industri Selatan V PP-7, Jababeka 2, Cikarang 17550, Indonesia; wangsa.ismaya@dexa-medica.com (W.T.I.); raymond@dexa-medica.com (R.R.T.); 2 School of Pharmacy, Bandung Institute of Technology, Ganesha 10, Bandung 40132, Indonesia; 3 Research Center for Nanosciences and Nanotechnology, Bandung Institute of Technology, Ganesha 10, Bandung 40132, Indonesia

**Keywords:** anticancer, biological active fraction, immunomodulator, sugar-binding proteins, therapeutic protein

## Abstract

The mushroom *Agaricus bisporus* secretes biologically active compounds and proteins with benefits for human health. Most reported proteins from *A. bisporus* are tyrosinases and lectins. Lectins are of therapeutic or pharmaceutical interest. To date, only limited information is available on *A. bisporus* lectins and lectin-like proteins. No therapeutic products derived from *A. bisporus* lectin (ABL) are available on the market despite its extensive exploration. Recently, *A. bisporus* mannose-binding protein (Abmb) was discovered. Its discovery enriches the information and increases the interest in proteins with therapeutic potential from this mushroom. Furthermore, the *A. bisporus* genome reveals the possible occurrence of other lectins in this mushroom that may also have therapeutic potential. Most of these putative lectins belong to the same lectin groups as ABL and Abmb. Their relationship is discussed. Particular attention is addressed to ABL and Abmb, which have been explored for their potential in medicinal or pharmaceutical applications. ABL and Abmb have anti-proliferative activities toward cancer cells and a stimulatory effect on the immune system. Possible scenarios for their use in therapy and modification are also presented.

## 1. Introduction

*Agaricus bisporus* is one of the most consumed edible mushrooms in the world, and its benefit to human health has been widely reported. The mushroom is a popular part of the human diet. It is commonly called white mushroom, button mushroom, or champignon. *A. bisporus* is rich in metabolites and other biologically active compounds (amino acids, simple sugar/saccharides, indole, phenolic compounds, fatty acids, sterols, statins, vitamins, trace elements, and minerals), complex carbohydrates, and proteins [1]. In total, the mushroom fruiting bodies contain on average (non-dried weight) ~30% proteins, ~35% carbohydrates, <5% others (sterol, saponin, tannin, terpenoid, minerals, and vitamins), and water (moisture) [2]. These constituents can be grouped into small and large molecules. The large molecules are proteins and complex carbohydrates. The mushroom is high in chitin content, thereby it is promoted for use as a source of dietary fiber. The small biologically active compounds and the proteins are associated with the anticancer, antiinflammation, antidiabetic, antihyperlipidemic, antioxidant, antiviral, and antimicrobial activities of the mushroom extract [1]. *A. bisporus* extracts inhibit the growth of epithelial and breast cancer cells as well as induce innate and adaptive immunity. Inclusion of the mushroom as part of the daily diet lowers the risk of breast cancer for postmenopausal women [3]. The anticancer activity is often referred to as an inhibition of aromatase activity by small compounds in the mushroom extracts or by immediate action of the lectin on the cancerous cells. These reports strengthen the attribute of the mushroom as a nutritional and remedial food.

The most studied proteins from *A. bisporus* are tyrosinase (also often called polyphenol oxidase, PPO) and lectin [4,5]. Indeed, bioactive proteins from edible mushrooms mostly comprise of lectin, ribosome-inactivating protein, copper oxygenase/oxidase (laccase, tyrosinase), antifungal proteins/peptides, and immunomodulatory proteins [6]. In this respect, it is important to note that ribosome-inactivating proteins are usually classified as lectin [7,8], which are known to display antifungal and immunomodulatory activities; hence, the antifungal and immunomodulatory proteins of the mushroom could also be grouped as lectin [9]. *A. bisporus* PPO and lectin are often employed in the search for human medicine, either as a bioactive molecule, as a molecular target, in the production of metabolites, or as a component in biochemical assays.

PPO catalyzes the conversion of L-tyrosine to L-3,4-dihydroxyphenylalanine (usually called L-DOPA), which is subsequently transformed into Dopaquinone, which is a precursor in melanin biosynthesis [4]. Melanin is a ubiquitous pigment in all organisms and PPO is associated with pigmentation in living organisms. PPO is mostly employed in the search for inhibitors against melanin formation, highly valuable in the search for skin whitening agents in the cosmetic industry and to prevent browning of agricultural produce [10]. The enzyme is also employed in the production of L-DOPA, the most effective agent in the treatment for Parkinson’s disease [11], which is caused by impaired pigmentation in the brain by PPO and PPO-related proteins [12]. However, the direct use of PPO in therapy has been challenged because of its potential toxicity. PPO suppresses tumor growth, but it is also mutagenic [10]. The PPO catalyzed reaction generates unstable Dopaquinone that leads to the production of harmful oxy radicals, peroxides, semiquinones, and quinones [13], which are responsible for the antitumor activity of PPO, but also are neurotoxic [10]. The toxicity of the products and adducts generated by PPO result in a reluctance to use the enzyme directly in human therapy. Mushroom tyrosinase is commercially available and has long been employed primarily as a potent and viable inhibitor for anti-pigmentation, but not for therapeutic uses. Therefore, PPO and PPO-related proteins are not included in this study.

On the other hand, lectin is a protein with hemagglutinating (agglutination of red blood cells) activity. Its activity originates from the ability to recognize and reversibly bind carbohydrate and glycoconjugate (glycoprotein and glycolipid) [9]. Lectin is not an immunoglobulin, but is highly selective in and specific to the type of sugar/glycan, interacting with bound and free glycan, including monosaccharides (such as glucose, mannose, and galactose). Its interaction with the glycan is similar to that between antigen and antibody or enzyme and substrate [14]. The lectin’s selectivity and specificity is the basis for its use in immunohematology, for example, as the marker for human blood typing [15]. The lectin family can be classified based on the structure of its carbohydrate recognition domain (CRD) or by the structural fold/feature. There are at least seven known groups of lectin: calnexin, L-type (β-sandwich), P-type (rich in β-structures), C-type (mixed α/β-structure), galectins (β-sandwich), I-type (immunoglobulin superfamily fold), and R-type (β-trefoil); the first three are intracellular, while the latter are extracellular [16]. The protein plays an important role in cellular processes, such as signaling, differentiation, and targeting [7]. Lectin is ubiquitous, but its occurrence and localization in tissues vary from one organism to another [17]. Most studies have been done with lectins from plants or animals, and only a limited number with lectins of fungal, bacterial, or viral origin. To date, only one lectin from *A. bisporus* has been reported, that is, *A. bisporus* lectin (ABL or ABA, *A. bisporus* agglutinin). Like mushroom tyrosinase, ABL is commercially available and has long been assessed for therapeutic use.

The use of *A. bisporus* in medication has mostly been studied using mushroom extracts, containing the small molecules (phenolic compounds, indoles, statins, and other secondary metabolites). For example, ergothioneine is an antioxidant associated with protection of monocytes activity, the indoles are associated with anticancer and anti-aging bioactivity, while ergosterol and ergocalciferol help to prevent vitamin D efficiency [1]. Commercial preparation of these compounds for therapeutic purposes includes complex extraction and isolation steps, while direct use of the extracts may be ineffective and undesirable. These small molecules are often present in a complex that is difficult to separate, whereas a long and complex extraction process may impact their stability and activity. Direct consumption of the fruiting bodies, that is, as part of the diet, might not be effective to achieve immediate therapeutic effect. On the other hand, the mushroom’s bioactive fraction containing ABL has been reported to increase both insulin secretion by the pancreas and calcium uptake by the pancreatic islets up to three- and eleven-fold, respectively [18]. Thus, it is commercially attractive to produce mushroom lectins using molecular biology, that is, heterologous expression of the lectins using microbial cells as the host. Using that approach, modifications to the protein molecule can be introduced to improve its characteristics.

This paper focuses on lectins from *A. bisporus*, including ABL, with special attention to Abmb (*A. bisporus* mannose-binding protein), a recently discovered lectin-like protein with a unique characteristic. On its own, Abmb has no agglutination capacity, but it can display agglutinating activity of lectin when it occurs in complex with the mushroom PPO [19]. Thus, Abmb is unique in its relation to the other *A. bisporus* lectins with the similar β-trefoild (ricin B-like) fold. Nevertheless, its biological activity allows its inclusion as a therapeutic protein.

To date, only few proteins from *A. bisporus* have been investigated experimentally using an active fraction isolated from the fruiting bodies or as a recombinant protein. They are ABL [5], Abmb [20], PPO1 and PPO2 [21], PPO3 [4,22], PPO4 [23], and the most recently reported AB21 [24]. The latter is stable at a wide pH range (4.5–10.5), is thermostable, and has an affinity to transition metal ions, but displays no lectin activity. The AB21 structure consists of a helix bundle organized as collagen-like [24]. The AB21 structure resembles the animal mannose-binding protein, but AB21 occurs as a dimer instead of trimer. The biological relevance of AB21 is unclear at this moment.

## 2. Lectins and Lectin-Like Protein in *A. bisporus*

ABL is commercially available, and can even be provided in biotinylated, fluorescein isothiocyanante (FITC), and other fluorescence label-conjugated forms, mostly for bioanalysis and diagnosis (detection) purposes. The protein was discovered over 50 years ago and its therapeutic potential has been extensively studied. Yet, there is no therapeutic protein product derived from ABL for human applications on the market. 

**Figure 1:**
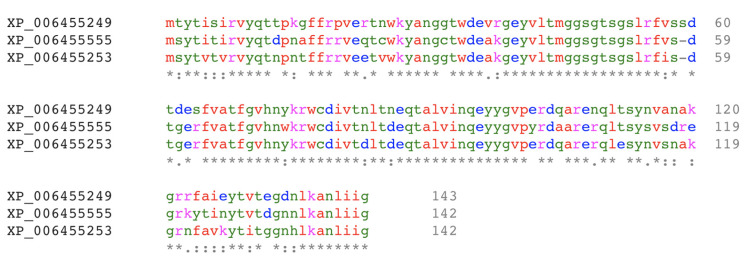
Alignment of amino acid sequences of *A. bisporus* lectin (ABL) (XP_006455249) and two of its closest putative lectin homologs in the *A. bisporus* U97 genomic library [27]. The protein annotations are according to the GenBank ID; the asterisk (*), colon (:), and period (.) signs indicate the identical, conserved, and similar amino acid residues, respectively.

To date, only limited information is available on the lectins from *A. bisporus*. Since the *A. bisporus* genome was sequenced in 2012 [25], only one lectin from *A. bisporus* has been registered in the Genbank (ABL, GenBank ID XP_006455249). In 1995, two cDNA coding for ABL were cloned and the identity of the four ABL subunits in the tetrameric form was revealed [26]. At that time, gene products’ isoformism was considered. Since the full genome of *A. bisporus* has been reported [25], no new lectins have been reported. The genomic data of *A. bisporus* H97 suggest the presence of 18 open reading frames (ORFs) that have been assigned as putative lectins. Two ORFs, Genbank ID of XP_006455555 and XP_006455253, were identified as *Xerocomellus chrysenteron* (red-cracking bolete mushroom) lectin (XCL) like proteins. The amino acid sequence of these two ORFs is highly homologous to ABL (Figure 1). However, as described later on (see 3.1), the isoformism of the subunits in ABL originates from variations in post-translational modification (glycoform), not from the merging of different gene products.

Next, two more ORFs, Genbank ID of XP_006460632 and XP_006461387, appear similar to lectin receptor kinase and legume-like lectin, respectively. Their similarity to the legume’s lectin is not a surprise, considering the close relationship of fungal and plant lectins [28]. Four ORFs (Genbank ID of XP_006462966: 361 amino acids, XP_006459007: 414 amino acids, XP_006459008: 274 amino acids, and XP_006458911: 423 amino acids) encode proteins that contain the Ricin B-like lectin fold at either their N- or C-terminal. Proteins that contain a lectin domain are called chimerolectin [29] and occur commonly, for example, the Ricin B-like lectin domain of the fungal *Marasmius oreades* [30] and the C-domain of the fruit fly *Drosophila melanogaster* lectin [31].

**Figure 2:**
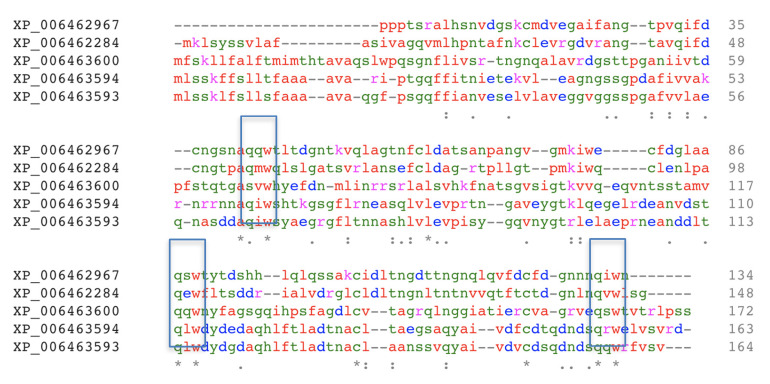
Alignment of amino acid sequences of the homologous putative Ricin B-like lectins [27]. The (Gln-x-Trp)_3_ canonical sequence motifs of β-trefoil fold are highlighted in boxes. The protein annotations are according to the GenBank ID; the asterisk (*), colon (:), and period (.) signs indicate the identical, conserved, and similar amino acid residues, respectively.

Finally, the amino acid sequences of ten ORFs contain structural features of the Ricin B-like lectin fold (Genbank ID of XP_006456463: 162 amino acids, XP_006455093: 186 amino acids, XP_006463600: 172 amino acids, XP_006462967: 134 amino acids, XP_006463594: 163 amino acids, XP_006463593: 164 amino acids, XP_006462284: 148 amino acids, XP_006463575: 164 amino acids, XP_006463601: 160 amino acids, and XP_006455522: 171 amino acids). The amino acid sequences of five of the ten putative Ricin B-like lectins are homologous (Figure 2). Ricin B-like lectin has a typical β-trefoil fold [32], which is composed of a three-domain assembly with a pseudo-symmetry orientation and contains a (Gln-X-Trp)_3_ sequence motif repeated in each of its domains [33,34].

On the basis of the carbohydrate binding module (CBM) database, lectins mostly contain CBM6, CBM13, CBM14, CBM18, CBM25, CBM26, CBM32, CBM36, CBM47, CBM57, and CBM60 [35]. In contrast, the *A. bisporus* genome indicates the potential presence of CBM1, CBM5, CBM13, CBM20, CBM21, and CBM35; where CBM1, CBM5, and CBM13 appear dominant [25]. CBM1 and CBM5 include lectins with affinity to complex carbohydrate, for example, cellulose [35] and possibly lignin [36], which is one of the major carbohydrate components in the mushroom. These CBM family members bind carbohydrates on the cellulose surface, so the binding site region in the protein surface is flat. Lectins from the CBM21 and CBM35 families bind to slightly less complex carbohydrate molecules, and have an affinity towards starches and xylan, respectively. Lectins from the CBM13 and CBM20 families bind oligosaccharides (four sugar units or more, non-branched) [35]. The CBM13 family includes lectin-like proteins and also binds short oligosaccharides (one to three sugar units).

Recently, a protein with a lectin-like structure was discovered [22] and named Abmb [37]. Interestingly, Abmb displays biological activities similar to lectins [20,38]. Abmb also has therapeutic potential [38]; hence, it is included in this review.

## 3. Agaricus Bisporus Lectin (ABL)

### 3.1. Morphology, Characteristics, and Genetics

In the early 1980s, ABL in mushroom extracts was found to be composed of two lectins, namely phytohemagglutinin-A and -B (PHA and PHB, respectively). Later, this mixture was found to contain four lectin isoforms (namely, I, II, III, and IV) with similar carbohydrate specificities and molecular weights [39]. ABL occurs as a ~60 kDa tetramer containing the four isoforms, presumably each weighing ~16 kDa. These isoforms have different pI values (5.53, 5.69, 5.98, and 6.70). Hence, they can be separated on a preparative isoelectric focusing chromatography column. The carbohydrate content analysis of the protein indicates that the four isoforms are glycoproteins [39]. The similarity of the four isoforms opens the possibility of variation in post-translational modification. Most importantly, the cDNA sequences of ABL isoforms obtained from a Southern blot analysis were identical. Further, the first 21 amino residues at the N-terminal of the four isoforms are also identical [26]. These results strongly suggest that the four ABL isoforms are derived from one gene product with variations in post-translational modification (glycoform). The isoforms with the most basic pI were successfully isolated and crystallized [40], which enabled the elucidation of the structure of ABL by X-ray crystallography.

ABL recognizes both galactose-β-1,3-N-acetylgalactosamine (known as Thomsen–Friedenreich antigen or T-disaccharide) and galactose-β-1,3-N-acetylglucosamine, but does not bind simple sugars (monosaccharides). ABL could also bind the sialylated form of the disaccharide (sialyl-2,3- galactosil-β-1,3-N-acetylgalactosamine) [41]. ABL agglutinates all erythrocytes independent of the blood group type. The type of sugar on the T-disaccharide recognized by ABL was characterized after treatment of erythrocytes with trypsin, which releases the O-linked glycopeptide, which completely abolishes ABL agglutination ability [39]. The ABL ability to agglutinate red blood cells originates from the presence of two distinct carbohydrate-binding sites on the ABL molecule [41], as confirmed by its crystal structure. ABL is classified as a hololectin, unlike R-type lectin (e.g., Abmb or its homologs), which is often found as a chimerolectin or merolectin [42].

**Figure 3:**
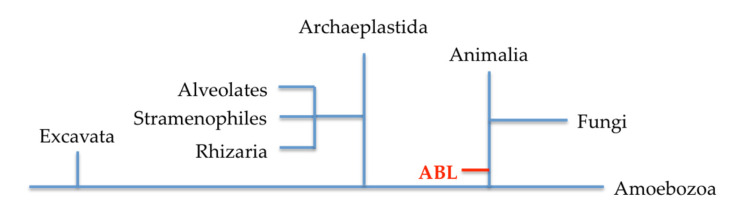
A phylogenetic relationship between fungal and plant lectins as presented by van Holle and van Damme [28].

From an evolutionary stand point, lectins with a similar structure to ABL are only found in fungi and archaeplastida from bryophytes (plants). The hypothesis is that the similarity of ABL with plant lectins is established via horizontal gene transfer upon endosymbiotics [28], thus fungal and plant lectins are evolutionarily related (Figure 3). However, the structural fold in ABL is unique and included as one of the twelve structural folds in plant lectins’ classification; in a newer classification for plant lectins, ABL is grouped as a lectin fold that is specific to certain plant phyla [42].

In the *A. bisporus* genome, two other genes code for proteins similar to ABL (Figure 1). The presence of multiple genes coding for protein isoforms in *A. bisporus* appears a common feature, for example, there are six PPOs in *A. bisporus*, namely PPO1–PPO6. PPO1 is expressed constitutively, while PPO2 is inducible [21]. PPO1 and PPO6 are located at chromosome 8, while PPO2–PPO5 are at chromosome 5. Expression of these PPO isoforms appears to be correlated with different stages in the development of the mushroom fruiting bodies and their compartmentation [4]. So far, the nature of ABL gene expression and its gene products’ compartmentation are not yet clear.

### 3.2. Structure and Possible Function in the Mushroom

**Figure 4:**
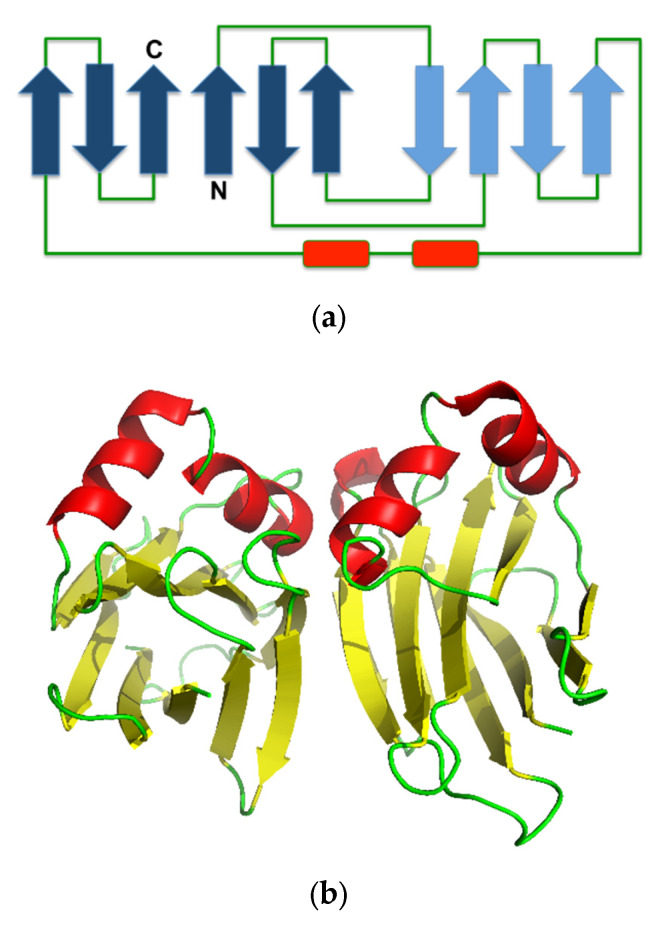
(**a**) Secondary structure topology of ABL. The pleated forms indicate the arrangement of the β-strands assembly. (**b**) Cartoon presentation of the crystal structure of ABL (Protein Data Bank (PDB) ID: 1Y2T). The structure was obtained from the Protein Databank and the three-dimensional structure figure was prepared using PyMoL [44]. The secondary structure topology was manually prepared using the three-dimensional structure as the reference.

The crystal structure of ABL contains a β-sandwich fold, consisting of uneven pairing of two pleated four and six β-strands with two helices inserted in the long loop that connects the pleated sheet, forming an α-loop-α motif (Figure 4a). The tetrameric form is composed of a dimer of an ABL dimer, which is assembled similar to actinoporin (Figure 4b) [7], a pore-forming toxin that is commonly found in fungal fruiting-body lectins [43]. The two carbohydrate binding sites in each monomer are located at opposite positions in the protein molecule, but the monomers are assembled in the tetrameric form in such a way that all binding sites are accessible to the solvent [41]. This assembly allows independent binding of two sugar molecules in each monomer. Hence, ABL can display multivalent sugar-binding, and thereby agglutination. Each of the two sugar-binding sites in the ABL monomer is unique because each can distinguish two ligands differing only in the orientation of one hydroxyl group (epimer) [31].

The binding of N-acetylgalactosamine occurs at the first binding site, which involves Tyr28, His72, Tyr74, Tyr98, and Arg107, while N-acetylglucosamine is coordinated by Asp79, Thr82, Ile102, Arg103, Tyr114, and Val116 at the second binding site. The residues involved in the binding of sugars are strictly conserved in other lectins that have actinoporin fold, such as XCL, *Neurosspora crassa* (red bread mold) lectin, *Pleurotus cornucopiae* (golden oyster mushroom) lectin, *Podospora anserina* (mold of herbivore faeces) lectin (41], and *Boletus edulis* (penny bun mushroom) lectin [45]. The alignment of the amino acid sequences of XCL, *N. crassa*, *P.cornucopiae*, and *P. anserina* lectins reveals the Gly-Val-**His**-**Asn**-**Tyr**-Lys-X-Trp-X-**Asp**-**Ile/Val**-X-**Thr/Val** motif. About 14 amino acid before the first Gly in that motif, there is a Gly-X-X-X-Leu-X-X-X-**Ser**-**Gly**-Thr-Ser-Gly-**Leu/Ile**-**Arg** motif. Moreover, about 14 amino acid residues after the last Thr/Val, there is a X-Tyr-**Tyr**-X motif. Five other sugar-binding residues are located close to either the N- (X-**Tyr**-**Ala**-Asn-Gly) or C-terminal (X-**Arg**-X-X-X-**Arg**-X-X-Gln-X-X-X-**Tyr**) (amino acid residues participate in the binding of galactosyl and glucosyl sugars are in bold). In the 142 long amino acid sequence of ABL, most amino acid residues, composing the first binding site, reside in the first 70 residues (N-terminal half), while the second binding site is at the C-terminal half. Interestingly, these sugar-binding residues are completely conserved in XP_006455253 (Figure 1), while in XP_006455555, the Tyr in the -His-Asn-Tyr- sequence motif is substituted with a Trp. However, it is unlikely that this substitution (Tyr74) alters the binding specificity or affinity, because this particular Tyr has minor contact with the sugar, providing a hydrophobic environment for the sugar ring to dock in the binding site [41]. Thus, so far, XP_006455555 and XP_006455253 could be predicted as the ABL isoforms with similar carbohydrate-binding activity.

Both ABL isoforms (GenBank ID of XP_006455555 and XP_006455253) are more basic (theoretical pI of 6.57 and 6.73, respectively; ABL is 5.85) and slightly more hydrophilic (GRAVY index of –0.542 and –0.531, respectively) than ABL (–0.529), likely because they contain less aliphatic side chains (Ala, Val, Ile, and Leu) (aliphatic index of 66.55 and 67.89, respectively; ABL is 68.11). This indicates that these putative ABL isoforms may be active in a different way in the mushroom or during development of the mushroom fruiting body. To date, very little is known about ABL function, on its compartmentation and localization of the encoding gene. Fungal lectins are postulated to function as a storage unit and/or a parasitic or infection agent. They also function in the development of fruiting bodies, morphogenesis, and defense mechanism. The latter is the most common proposed function, considering fungal lectin’s nematodicidal and insecticidal properties [45].

## 4. *Agaricus bisporus* Mannose-Binding Protein (Abmb) 

### 4.1. Morphology, Characteristics, and Genetics

Abmb was discovered as the light subunit of mushroom tyrosinase (PPO3) upon elucidation of the structure of the enzyme [22]. Abmb had been found consistently as an intrinsic part of the enzyme tetrameric complex [46], although its identity as a tyrosinase gene product had been questioned [47]. So far, separation of Abmb from the enzyme has not been successful, suggesting a strong and stable protein complex. Most importantly, this assembly of two proteins with different activity may indicate their synergistic functions in the mushroom.

Abmb is a non-glycosylated protein and migrates as an ~18 kDa species in a size exclusion chromatography column [38] (theoretical molecular weight of 16.5 kDa), thus in solution, it is monomeric. The protein has a pI value of ~4.9, as judged from an isoelectric focusing electrophoresis analysis [38] (theoretical pI is 5.34, thus, in actual condition, many of the negatively charged residues are buried). Abmb has a melting temperature at 50 + 1°C and appears unstable at pH below 2.0. However, the protein resists proteolytic degradation by trypsin and papain, but not by bromelain [38]. During an absorption capability test using fresh jejunum, the full-length Abmb was digested, resulting in a ~14 kDa species in an SDS PAGE analysis [38]. Interestingly, this ~14 kDa species resembles the Abmb molecule that occurs as the tetrameric complex with PPO3. This tetrameric complex is consistently obtained from the commercial preparation, which is directly extracted from the mushroom fruiting bodies [22].

**Figure 5:**
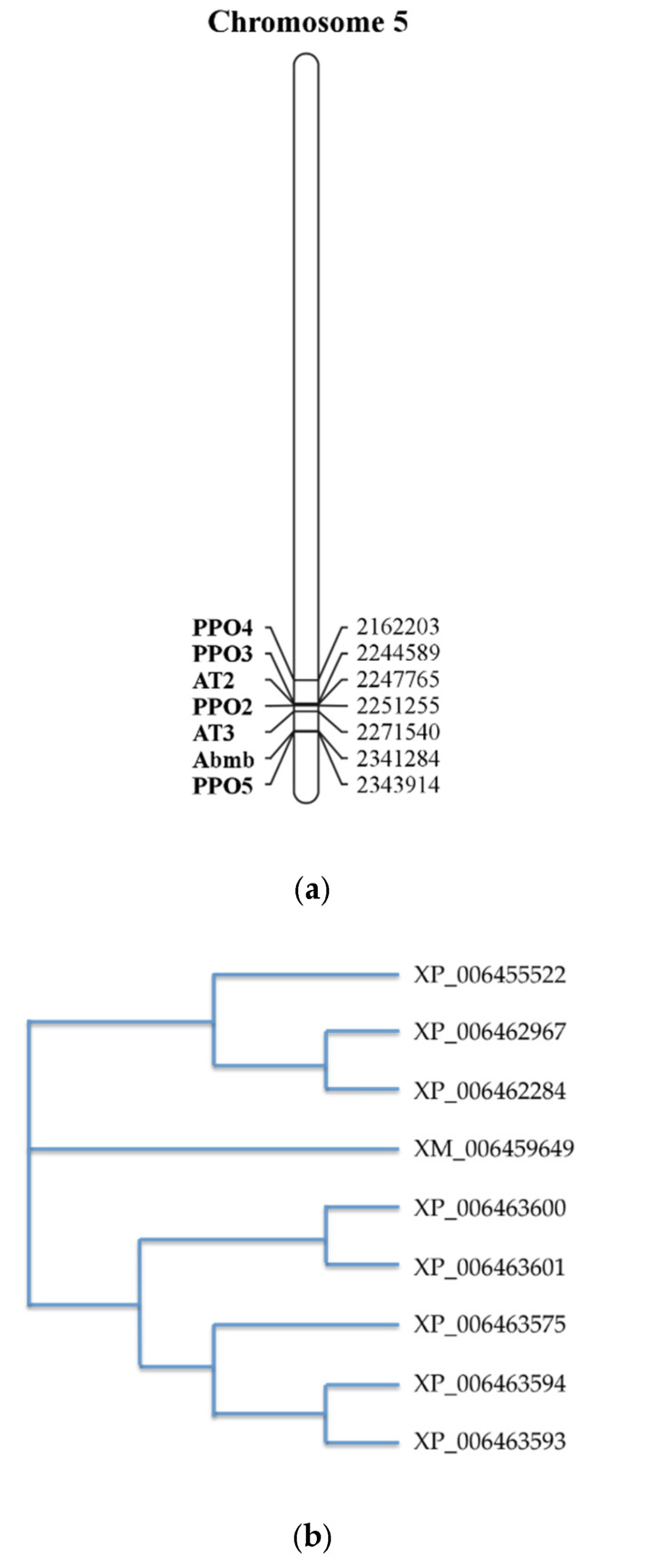
(**a**) The gene assembly in the region surrounding the Abmb encoding gene [4]. Numbers on the right refer to the position of the gene in the nucleotide genome sequence. AT stands for (4-hydroxy)phenylpyruvate amino transferase (**b**) Phylogenetic relationship between Abmb (GenBank ID XM_006459649) with other putative Ricin B-like lectins in *A. bisporus*. The phylogenetic tree was generated upon the amino acid sequence alignment using the program ClustalOmega [27]. PPO, polyphenol oxidase; Abmb, *A. bisporus* mannose-binding protein.

The Abmb encoding gene is located at chromosome 5, together with PPO (Figure 5a) [4]. Interestingly, despite its complex formation with PPO3, the location of the Abmb encoding gene sequence is closer to PPO5, one of the mushroom tyrosinase isoforms. The two genes are in opposite directions upon their translation. Abmb’s highest gene expression occurs during the mycelium phase, early development of the flesh (stage 3), and development of the skin (stage 3–7). This Abmb gene expression correlates closely with that of PPO5. This is not a surprise considering their close co-localization on the chromosome [4].

Abmb was not recognized as a lectin upon elucidation of the *A. bisporus* genome [25]. During an amino acid sequences alignment of ten ORFs coding for the putative Ricin-B like lectins, eight ORFs are similar Abmb. A phylogenetic tree analysis suggests that Abmb is not closely related to these eight ORFs. This indicates that Abmb may have a different function or activity from the eight ORFs. The two closest homologous ORFs to Abmb are XP_006463575 and XP_006455522, but their homology scores to Abmb are below 20%. The sequence analysis even suggests that the Abmb structural homologs from the mushroom *Clitocybe nebularis* and the bacterium *Clostridium botulinum* are more closely related. This may be related to the sugar-binding characteristics of Abmb, because Ricin B-like lectins (lectins with β-trefoil fold) are galactose-binding proteins [48].

Most importantly, during a screening for sugar affinity by surface plasmon resonance analysis, Abmb was found to bind specifically to mannose, without any affinity for glucose, galactose, or fructose [37]. This result was confirmed by an in vitro analysis using MCF-7 and MDA-MB231 breast cancer cells. Abmb appears to be monomeric at higher concentrations and does not display the agglutination ability of lectin (tested up to 500 ppm ~30 mM) [49], which is plausible as lectins with Ricin B-like structures with agglutination activity are oligomers in a complex with other proteins, or have a multivalent sugar-binding capacity. Abmb is predicted to have only one sugar-binding site that is similar to HA-33 and CNL, which are Abmb’s closest structural homologs (see below) [50]. Recently, it has been demonstrated that Abmb participates in the agglutination of red blood cells when it occurs as a complex with the mushroom tyrosinase [19]. This strengthens the hypothesis that Abmb and PPO3 activities are synergistic and serve one function in the mushroom fruiting bodies. Most importantly, in the tetrameric complex with PPO3, Abmb is present in a mature form, in which one of the surface loops is proteolytically cleaved. The fact that Abmb is still able to cause agglutination suggests that the maturation has no impact on its sugar-binding capacity.

### 4.2. Structure and Possible Function in the Mushroom

The crystal structure of Abmb (PDB ID 5EHA) contains twelve β-strands organized in three domains, each of which contains two antiparalel β-hairpins. The Abmb domain architecture resembles that of Ricin B-like lectin, in which the first domain consists of β1, β2, and β3 of the N-terminus and β12 of the C-terminus, forming a classical Greek key motif. This is in line with the fact that Abmb’s closest structural homologs are the hemagglutinin components of *C. botulinum* toxin (HA-33) and the Ricin B-like like lectin from *C. nebularis* (CNL) [22]. These two Abmb structural homologs share a similar carbohydrate-binding region and have a monovalent sugar binding mode; thus, the carbohydrate binding site of Abmb was postulated to resemble them [50]. However, HA-33 and CNL bind galactose; thereby, the sugar-binding fashion might differ, as demonstrated by a variation in the ABL sugar-binding sites. Indeed, the β-trefoil fold has been suggested to originate from a gene fusion event that links a 40-ish amino acid peptide with galactose-binding activity; each of these peptides becomes the subdomain [32]. Nevertheless, based on a phylogenetic study (Figure 5), Abmb is not closely related to any of the putative Ricin B-like lectins in *A. bisporus*.

**Figure 6:**
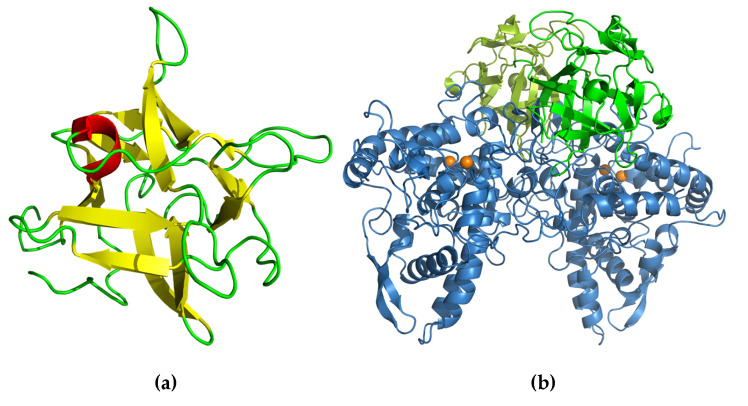
Cartoon presentation of the crystal structure of (**a**) Abmb (PDB ID: 5EHA) and (**b**) the tetrameric complex of PPO3–Abmb (PDB ID 2 × 9Y). In the PPO3–Abmb complex, the tyrosinase is at the bottom, while the Abmb molecules are at the top. The structure was obtained from the Protein Databank. The three-dimensional structure figure was prepared using PyMoL [44].

The structure of monomeric full-length Abmb [51] shows very little deviation from the protein structure in the complex with PPO3 (Figure 6) [22]. A structural arrangement occurs at the N-terminal part, in which the flexible N-terminal rotates 90° to expose an Abmb region that interacts with PPO3. The structure of the monomeric Abmb also shows the flexibility of a surface loop that is missing in the structure of Abmb in complex with PPO3 [51]. The sugar-binding in Abmb is similar to CNL, but the soaking of Abmb crystal with lactose, glucose, raffinose, or sucrose suggests that no interaction takes place with these sugars. The Abmb recognition of mannose (and mannitol), but not glucose (nor sorbitol), fructose, and galactose [37], provides a good explanation for the failed attempts to obtain Abmb crystals that contain lactose or glucose. Currently, an elucidation of the structure of Abmb in the presence of mannose and mannosyl sugars is on the way.

The function of Abmb in the mushroom is not yet clear. However, the recent finding that Abmb in complex with PPO3 can execute agglutination may provide a hint on its biological function in the mushroom fruiting bodies [19]. A similar situation occurs for HA-33, which can only bind one sugar, but can display agglutination when in a hemagglutinin complex with HA-17, as part of the larger botulinum toxin complex containing HA-13 and HA-70 [52]. Thus, in mushrooms, assigning a lectin function to Abmb appears appropriate. PPO has an important role in the biosynthesis of melanins [4], which are bacteriostatic. Hence, the enzyme may play a role in a defense mechanism [53]. Abmb and PPO3 would be a synergic protein complex in the mushroom’s defense mechanism. Abmb is most expressed during the mycelium stage and its presence remains high during the development stages of the fruiting bodies. Moreover, PPO5 is highly expressed in the mycelium stage and downregulated during the development of the fruiting bodies. PPO3 expression is regulated opposite to that of PPO5, thus PPO3 expression might compensate for the decreasing PPO5 expression [4]. It is possible that, during the transition from PPO5 to PPO3 and the down and up regulation of their expression, both PPO3 and Abmb undergo maturation and form the Abmb–PPO3 complex. Recruitment of PPO3 to form a complex with Abmb appears an obvious mechanism, because gene expression of PPO3 occurs in all fruiting body tissues (stem, flesh, and skin), except for in the gills (where PPO4 is present instead), at all development stages, ensuring that Abmb is present in most tissues. This hypothesis emphasizes the crucial role of Abmb in the mushroom defense mechanism. It is important to note that Abmb specificity towards mannose and mannitol (presumably also to mannosyl sugars) may complete the defense mechanism because the other lectins with β-trefoil in the mushroom *A. bisporus* are presumably galactose and/or glucose (and their derivatives) binding proteins.

## 5. Potential Therapeutic Application of Lectin and Lectin-Like Protein from *A. bisporus*

### 5.1. ABL

The potential use of ABL has been studied extensively for various therapeutic purposes. The first and best-known biological activity of ABL is its ability to inhibit proliferation of cancerous human epithelial colon cells (HT29) in vitro at a very low concentration (50 μg/mL ~0.8 μM) [54]. The activity of ABL on colon cancer cells is likely derived from its ability to block the import of protein into the nucleus required for DNA synthesis during cell proliferation, directly blocking internal protein transport at the nuclear pores [5]. ABL also inhibits MCF-7 (breast cancer cells) and Caco-2 cancer cell proliferation in vitro, although at a lower potency [54]. Furthermore, the introduction of ABL to cultured human keratinocytes and papilloma transformed cells results in lower cell growth [55]. ABL slows down proliferation of human ocular fibroblast and reduces collagen lattice contraction in vitro, which is beneficial for wound healing of glaucoma patients after a trabeculectomy procedure [56]. At 90 μg/mL (~1.5 μM), ABL suppresses proliferation of retinal pigment epithelium (RPE) cells, which subsequently lowers the proliferative vitreoretinopathy and prevents redetachment of retinal cells after a surgical detachment procedure [57]. Moreover, exposure of rat’s islet of Langerhans to ABL increases the conversion of pro-insulin to insulin, by enhanced proliferation of the pancreatic β-cell in vivo [58]. Thus, ABL might be employed in therapy for type-1 and -2 diabetic patients who are suffering from a damaged pancreas. Finally, ABL also shows a strong inhibitory activity against human immunodeficiency virus type-1 (HIV-1) reverse transcriptase (IC50 of 8 μM) in vitro [59]. These reports show that ABL has a wide variety of cell targets and can be exploited for therapeutic applications as an anticancer, antidiabetic, and antiviral agent.

ABL has no cytotoxicity to normal cells [5]. However, ABL suppresses the immunoglobulin production in T and B lymphocyte cells through inhibition of DNA synthesis [60]. The introduction of ABL to the macrophage cells of mice, after stimulation with lipopolysaccharides, induces nitric oxide and the production of tumor necrosis factor (TNF)-α, both in vitro and in vivo [61].

### 5.2. Abmb

Abmb can permeate a dialysis bag made of fresh jejunum ex vivo [20], and this ability is not altered upon its bioconjugation with a drug model [62]. This suggests that the protein might be employed as a drug carrier for oral administration. The use of Abmb as a drug carrier is supported by its resistance to the harsh gastrointestinal tract conditions [38]. Although Abmb internalization by the epithelial monolayer barrier of the intestine has not been described, generation of a species similar to mature Abmb (as in complex with PPO3) [38] indicates that Abmb undergoes proteolysis during the absorption, opening the possibility of transcellular permeability. In general, lectin absorption in the intestinal monolayer barrier is considered to occur via trans- or endocytosis [63]. At this moment, the mode of Abmb internalization by the intestinal cells is being studied.

At 12.5 μM, Abmb inhibits proliferation of MCF-7 breast cancer cells and arrests growth at lower concentrations in vitro [38]. The effect of Abmb administration at lower concentrations on MCF-7 cells proliferation does not impact the cell cycle, nor cell death. At higher concentrations, cell apoptosis occurs, as indicated by an increased expression of caspase-3, caspase-8, and caspase-9; upregulation of the pro-apoptosis protein Bcl-2-associated X protein BAX; and downregulation of anti-apoptosis protein B-cell lymphoma-extra large Bcl-xL. Moreover, at low concentrations, Abmb activates expression of p53, which is important for genome stability by preventing genomic mutations [38]. At concentrations up to 6.25 μM, Abmb slightly increases proliferation of RAW 264.7 macrophage cells. Thus, the protein could potentially be recruited as an immunostimulator.

A preliminary immunogenicity test in Swiss Webster mice suggested that Abmb does not evoke a response of the immune system [20]. Further toxicity and immunogenicity tests with the inbreed and outbreed mice showed that Abmb was safe after prolonged administration at an elevated dose [64,65]. These results strengthen the safety profile of Abmb. This safety assessment is important for future use of Abmb.

### 5.3. Potential Therapeutic Application from the Perspective of Other Lectins

Lectins of plants, fungi, archaea, actinomycetes, and bacteria have been studied for their application as antiviral agents [66]. Lectin’s potencies as an antiviral agent have been shown against a variety of viruses, for example, herpes simplex type 1 and 2, hepatitis C, influenza A/B, HIV, Japanese encephalitis virus, and the current Coronavirus Covid-19; the effective concentration range is 1.6 nM–1.3 μM (see [66] and the references therein). The potential use as an anticancer agent has also been widely studied with various cancerous cells such as epidermal keratinocyte, lung, thyroid, [67], fibroblast, colon, prostate, colorectal, melanocyte, liver, mesenchyma, lymphoid, brain, and spinal (and the references therein). Most of these potentials therapeutic applications have been tested with ABL (see above), but not with Abmb, because of its ambiguous function and activity.

## 6. Future Development of Abmb

ABL is a potent anticancer agent, but its development as such has halted in the last decade. Instead, other possible applications in diabetes therapy or modulating the immune system emerged. The concentration of Abmb required to produce a significant effect on cancer cells is much higher than with ABL. Thus, Abmb development as an anticancer agent may require further assessment and possibly modification of the protein molecule.

**Figure 7:**
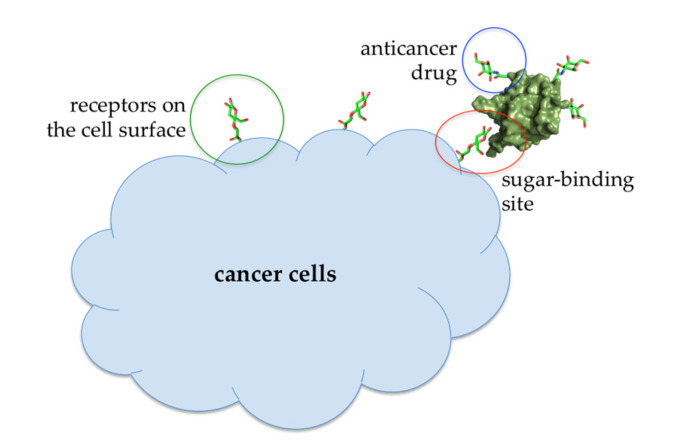
Illustration of an Abmb bioconjugate carrying an anticancer drug upon recognition by a receptor (presumably a sugar) on the cell surface.

At lower concentrations, Abmb might be useful for other purposes, that is, targeted drug carrier or complementing the immune system, taking the advantage of Abmb’s (and its bioconjugate) ability to permeate the jejunum and its specific affinity to mannose, respectively. Abmb could be conjugated to an anti-breast cancer drug, making sure the drug is directed to the cancer cell target (Figure 7). Upon attachment to the receptor on the cell surface, the anticancer drug load could be released and Abmb goes on to circulate in the blood stream, or the bioconjugate as a whole undergoes endocytosis. The latter is a plausible proposition based on Abmb’s ability to initiate p53 expression. In this instance, harnessing p53 role in cell apoptosis and senescence has been considered as the most useful cancer therapy [68]. Therefore, elucidation of Abmb’s exact mechanism in activating the p53 expression is crucial. The strategy to bring an anticancer drug into the cell target has been extensively developed. As an example, mannose, decorated on the surface of nano particles containing doxorubicin, has been successfully demonstrated to deliver the drug into breast cancer cells [69]. This strategy exploits high expression of mannose receptors on the surface of human malignant tumor cells. Though the nature of Abmb interaction with the breast cancer cells is not yet known, the Abmb specificity towards this type of cancer cell could be exploited.

Interestingly, breast cancer chemotherapy using the combination of doxorubicin and cyclophosphamide is reported to have a negative impact because the mannose-binding lectin 2 (MBL2) expression in breast cancer patients receiving this combination of drugs is downregulated [70]. Lower expression of MBL2 leads to higher risk of grade 3 infection from a compromised immune system condition. Introduction of Abmb into the blood circulation might compensate downregulation of MBL2, slow down breast cancer cell growth, and stimulate the immune system. Abmb could be administered via oral administration, benefiting from its absorbability in the intestine. Recently, a strategy to deploy magnetic beads coated with the fusion of the MBL and Fc domain of the antibody has been successfully developed to capture circulating tumor cells, mimicking the opsonization mechanism of the innate immune system [71]. This strategy could be adopted to further Abmb functionalization.

## 7. Conclusions

Lectins and lectin-like protein from the mushroom *A. bisporus* hold a great potential for application in health and medicine. Lectins can be consumed as part of the diet or economically produced either as protein extracts (from cheaply and easily cultivated mushroom) or as recombinant proteins for specific targeting in medicinal or pharmaceutical application.
